# ANALYSIS OF THE URBAN HEAT ISLAND IN ŁÓDŹ, POLAND

**DOI:** 10.13075/ijomeh.1896.02458

**Published:** 2024

**Authors:** Monika Bobrowska-Korzeniowska, Joanna Jerzyńska, MaŁgorzata Paciorek, WŁodzimierz Stelmach

**Affiliations:** 1 Medical University of Lodz, Department of Paediatrics and Allergy, Copernicus Memorial Hospital , Łódź, Poland; 2 Office of Proecological Studies and Measurements “Ekometria”, Gdańsk, Poland; 3 Poddębice Health Center, Poddębice, Poland

**Keywords:** health effects, urban heat island, UHI, Łódź, UHI index, UHI contrast

## Abstract

**Objectives:**

The phenomenon of urban heat island (UHI) is based on the occurrence of elevated air temperature in urban areas, in relation to the surrounding urban, rural and forest areas. The aim of the study was the assessment of the UHI in Łódź in 2014–2019. Łódź is a large city with an area of 293.3 km^2^, located in the center of Poland.

**Material and Methods:**

The UHI was defined as the difference of at least 1.5°C in daily minimum temperature at the point representing the commutative conditions (lower temperature) and daily minimum temperature at the point located in the city center (higher temperature). Based on data from the Weather Research and Forecasting (WRF)/CALMET models and the above criterium, the occurrence of UHI cases was analyzed.

**Results:**

In summary the phenomenon of UHI in 2014–2019 was observed in every studied year, most often in 2018 with the highest UHI index almost 5°C, and increased over the years. The results proved that the highest UHI contrast was seen at night between 11 p.m. and 3 a.m.

**Conclusions:**

The study confirms that the UHI phenomenon in Łódź is persistent and has expanded in spatial extent over the years due to urban growth.

## INTRODUCTION

The phenomenon of urban heat island (UHI) is based on the occurrence of elevated air temperature in urban areas, in relation to the surrounding urban, rural and forest areas [1,2]. It is dynamic in time and is characterized by high variability both daily and annual, and is associated with the type of weather, specially formed under the influence of local conditions. In dynamic weather conditions, at high wind speed, cloud cover and precipitation, UHI does not occur. Urban heat island is particularly intense in the evening, night and morning, when the temperature in the city can be up to several degrees Celsius (°C) higher than outside the city, however, during the day, as a rule, the phenomenon disappears. The dependence of the intensity of the phenomenon on the size of the city is observed [3]. It is estimated that with a population of 500 thousand −1 million, the air temperature in the city is usually higher by 1.1–1.2°C than outside the city.

Stabilization of the thermal balance is facilitated by the participation of natural plant surfaces. Unfortunately, most of the city is covered by impermeable surfaces, which reduces evaporation from soil and vegetation, and thus increases the temperature [4].

Covering the surface of streets, pavements, squares with impermeable materials (asphalt, concrete) also causes increased outflow of rainwater. Only a small part of the water evaporates, which in turn results in a lower content of water vapor in the air in the city and reduced heat dissipation as a result of evaporation.

Another element is the modification of the heat balance of the city by human activity, which consists primarily of heat produced by heating and air conditioning devices, but also industry or car traffic. The phenomenon of UHI indicates that in addition to changes in climatic conditions in the city, it has several negative health effects. It was found that an increase in temperature by 1°C may cause an increase in the mortality rate by 1.84–3.12% [5]. The main one is the increase in heat stress, which contributes to the appearance of specific physiological reactions resulting in specific symptoms such as heat fainting, heat cramps, thermal edema, exhaustion or heat stroke [6–8]. High temperatures affect the course of cardiovascular and chronic respiratory diseases and also have an indirect effect on the exacerbation of symptoms of respiratory allergic diseases by extending the pollen period and increased allergenicity of pollen [9,10].

Łódź is a large city with an area of 293.3 km^2^ located in the centre of Poland [11]. Together with the cities directly adjacent to Łódź, it forms an agglomeration with a population of almost 1 million. Łódź is located on a relatively sparsely varied terrain, away from large water bodies and larger rivers or other geographical units that could modify local climatic conditions. The prevalence of allergic diseases in Łódź is high, according to epidemiological studies almost 40% of Polish urban population may suffer from allergic rhinitis and about 20% from asthma [12].

In Łódź, historical data on the differences in the minimum temperature in 1934–1936 between the station in the city center and a local station indicate the existence of a well-established UHI with a clearly marked annual cycle [13,14]. A very similar result was observed regarding the maximum temperature differences between the city center and local station [15,16]. In the summer UHI in Łódź usually reached 3–6°C. During the winter, the thermal contrasts of the city-terrain rarely exceeded 2–3°C. However, in the winter, in the case of advection of cold, arctic air over a heated city, there were extremely large temperature differences. The aim of the study was the assessment of the UHI in Łódź in 2014–2019.

## MATERIAL AND METHODS

The analysis of the UHI phenomenon was based on data from Weather Research and Forecasting (WRF) Model for the years 2014–2019 [17]. Calculations using WRF Model were made in 2 nested grids, the first with a resolution of 15 km covering a large part of Europe and the second with a resolution of 5 km covering the area of Poland with a margin of approx. 250 km. In order to refine, data from WRF Model were processed using the CALMET preprocessor to 1 km resolution [18].

**Figure 1.**
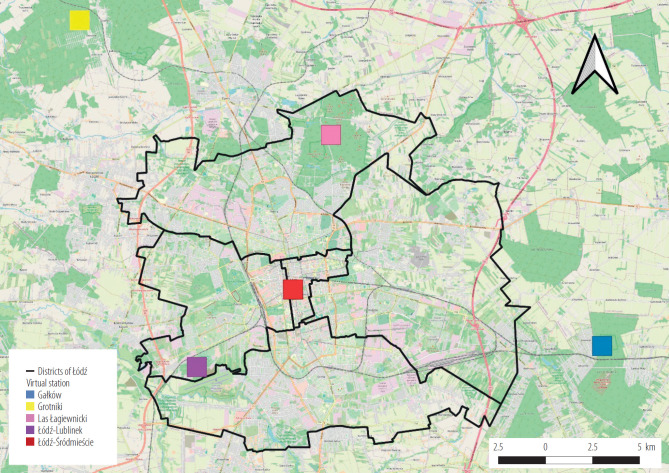
Location of virtual stations (temperature assessment points) to assess the urban heat island (UHI) in Łódź, Poland, 2014–2019

Urban heat island index and the contrast criterion were adopted as basic indicators determining the UHI phenomenon. The UHI index was defined as the difference of at least 1.5°C in the daily minimum temperature between a point representing suburban conditions (lower temperature) and a point located in strictly urban conditions (higher temperature), such as the city center [19,20]. The UHI contrast criterion was defined as the occurrence of a temperature difference of at least 1.5°C, for a single hour in the day, between the warmest point in the urban area and a selected point in the suburban area. This criterion allows the determination of UHI instances [19,20]. Based on data from the WRF/CALMET models and the above measures, the occurrence of UHI cases in Łódź in 2014–2019 was analysed. For the analysis, 5 single grids from CALMET meteorological data was chosen. Each grid represents virtual stations with different thermal characteristic. For those grids hourly temperature values were plotted and then the above-mentioned indicators were calculated (Figure 1).

Characteristic of virtual stations :

1. Łódź-Śródmieście: located in the dense residential area in the city center, representing the highest temperature.
2. Łódź-Lublinek: corresponding to the synoptic station at the airport, representing suburban conditions with dispersed building development.
3. Las Łagiewnicki: located in the north of the city in the Bałuty district, representing suburban conditions influenced by forested land.
4. Gałków: located in the southeast, representing rural conditions outside the borders of Łódź.
5. Grotniki: located in the northwest, also representing rural conditions outside the borders of Łódź.

Analyses of information on the temperature distribution in Poland presented annually in the Polish climate monitoring bulletin clearly indicate climate warming [21]. This may potentially affect the increase in the frequency of UHI phenomenon and the deepening of heat stress in cities. The years 2014–2019 in the region of city of Łódź were very warm and the years 2014, 2015, 2018 and 2019 were even extremely warm [21]. This was confirmed by the average annual temperature distributions in Łódź determined from the WRF/CALMET models, where yearly mean temperature was 1–2°C higher than in 1971–2000 (for example in 2019 yearly average temperature in Łódź was about 10°C, when typical temperature in 1971–2000 for this area was about 8°C) [22,23].

## RESULTS

### Analysis of the UHI index in Łódź in 2014–2019

In 2014, a total of 74 UHI >1.5°C episodes were recorded, of which the most common difference was recorded between Łódź-Śródmieście (city centre) and Gałków (rural area) points. The largest difference in minimum temperature was 4.3°C and was recorded in rural area of Grotniki (July) and Gałków (February). Within the city limits, this difference was 4.1°C in Las Łagiewnicki and 3.8°C in Łódź-Lublinek point (suburban). The phenomenon of UHI appeared quite evenly during 2014.

Although the UHI index was more frequent in 2015, it was more often 1.5°C, in general the temperature differences were lower. In 2015, a total of 86 UHI >1.5°C instances were recorded, of which the most common difference was again recorded between Łódź-Śródmieście (city centre) and Gałków (rural area) points. There was also the largest difference in the minimum temperature, which was 3.5°C. Within the city limits, this difference was 3.2°C in Las Łagiewnicki (forested area) and in Łódź-Lublinek (suburban) points. Most often, the phenomenon of UHI appeared in spring and autumn.

In 2016, the UHI index was the rarest to take >1.5°C values, as only 49 occurrences were recorded. However, the maximum temperature differences between Łódź-Śródmieście (city centre) and Gałków (rural area) and suburban area (Łódź-Lublinek) did not exceed 3.5°C and within the city 3°C. The phenomenon of UHI appeared most often in summer and spring.

In 2017, the UHI index in the analysed points assumed 57 times the values of >1.5°C. The maximum temperature differences between city centre (Łódź-Gałków) and the rural area were marked in Gałków point and they exceeded 4°C. Within the city, the larger UHI index assumed higher values in Las Łagiewnicki (3.6°C) than in Łódź-Lublinek (2.4°C) point. Most often, the phenomenon of UHI appeared in winter and autumn.

The year 2018 was characterized by the most frequent occurrence of UHI >1.5°C in the analyzed points. As many as 129 episodes were recorded, most often in Gałków point (rural area), and least often at the position in Łódź-Lublinek point (suburban area). The maximum temperature differences between the city centre and the suburbs were 4.7°C. Within the city, these differences were about 3.5°C. Most often, the UHI phenomenon appeared in summer and autumn.

The year 2019 was by far the warmest among the analyzed. While the number of UHI >1.5°C occurrences in the ana lyzed points was not the largest (122 occurrences were recorded), the index itself took the highest values. The maximum temperature differences between the city centre and suburbs exceeded 4–5°C. Within the city, the differences in the minimum temperature were smaller and amounted to about 3°C. The UHI phenomenon appeared practically throughout the year, but the largest temperature disproportions concerned the winter period.

**Figure 2.**
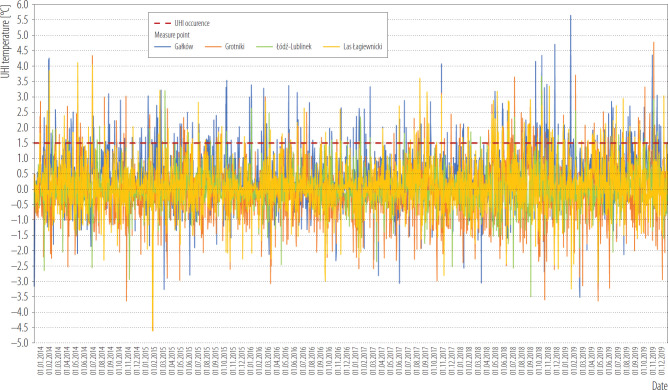
Urban heat island (UHI) index in 2014–2019 for selected points in Łódź, Poland

In summary the phenomenon of UHI in 2014–2019 was observed in each analyzed year, of total 517 times. In the analysed period, the UHI phenomenon most often appeared in winter and autumn. The year with the most frequent occurrence of the UHI phenomenon was 2018, then it was also the most intense (Table 1, Figure 2).

**Table 1.** The annual changes of urban heat island (UHI) episodes in Łódź, Poland, 2014–2019

Variable	2014	2015	2016	2017	2018	2019
UHI episodes [n]	74	86	49	57	129	122
Maximum temperature difference [°C]	4.3	3.5	3.4	4.1	4.7	5.7
Most frequent						
season	spring (N = 20)	winter (N = 18)	spring (N = 10)	summer (N = 15)	autumn (N = 32)	autumn (N = 25)
month	February, March (N = 8)	October (N = 9)	March (N = 6)	August (N = 9)	October (N = 13)	November (N = 12)

### Analysis of the UHI contrast criterion in Łódź in 2014–2019

The occurrence of differences of 1.5°C between the analyzed points in individual hours indicates the probability of UHI phenomenon occurs. This shows its frequency and diurnal cycle.

Most often in 2014, the temperature contrast was marked in Gałków point (rural area) The months with the potentially most common UHI phenomenon were April and July (about 25 appearances in each of these month). Same situation was in 2015 but months with the potentially most common UHI phenomenon were September and June (about 30 appearances in each month).

A minor difference in 2016 and 2017 compared to 2014 and 2015 was the increase in the number of UHI occurrences in Grotniki (also rural area). Months with the potentially most common UHI phenomenon in 2016 were May and September (about 15 appearances in each of both months) and in 2017 August and September (about 30 appearances in each of both months).

In difference to previous years, in 2018 UHI contrast has often been also observed in Las Łagiewnicki point (suburban forested area). In all above-mentioned points, potential phenomenon of UHI could be observed in May–July (>40 appearances) and September–November (about 40 appearances). A similar situation was recorded in 2019, however months with the potentially most common UHI phenomenon are April, June and July (>30 appearances). In period of 2014–2019 the appearance of UHI contrast was least often in Łódź-Lublinek.

Analysing the daily distribution of temperature UHI contrast in period 2014–2019, it can be concluded that the greatest differences occur at night between Łódź-Śródmieście (city center) and the Gałków (rural area). During the day, this disproportion is small or disappears. The differences between city center (Łódź-Śródmieście) and Łódź-Lublinek (suburban area), although they are marked, are small. In the city, much lower temperatures characterized forest areas, which clearly indicates the significant impact of green areas improving thermal conditions in the city. The highest contrast was noted in nighttime at 1:00–3:00 a.m.

**Figure 3.**
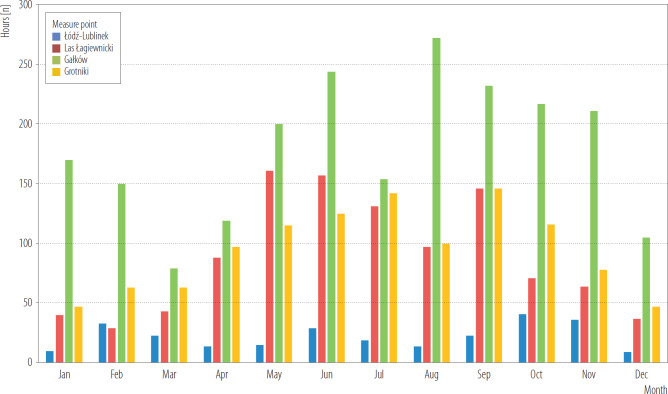
Frequency of hours with contrast within months of 2014–2019 between Łódź city centre and suburban areas, Poland

**Figure 4.**
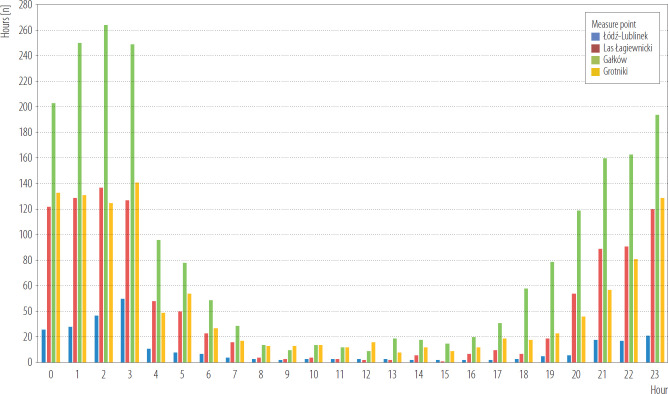
Frequency of urban heat island (UHI) contrast between Łódź city centre and suburban areas during the day in 2014–2018, Poland

In summary, in 2014–2019 UHI contrast of temperature was observed in different months in April–November. The most pronounced UHI contrast were observed during nighttime hours at 1:00–3:00 a.m. (Figure 3 and 4).

## DISCUSSION

Santamouris et al. [24], Akbari [25] and Fortuniak [16] noted that the formation of the UHI is the result of the synergy of many physical processes, of which the most important role is played by: the radiation balance of the city, the increased thermal capacity of building materials, anthropogenic heat flux, reduced evapotranspiration, reduced turbulent heat transport. All these elements are related to the structure of land use in the city, including with particular emphasis on the type of development.

The climatic conditions of Łódź, like the whole of central Poland, are shaped by the masses of polar-sea and continental air, which makes the climate transient, it is expressed by frequent changes in weather conditions and the occurrence of 6 seasons. A significant impact on the individual components of the climate of Łódź has its location within and at the foot of the Łódź Hills, which modify the speed of winds and the amount of precipitation reaching the city. Łódź receives the largest in central Poland amount of precipitation − of the order of ≥600 mm [22,23].

The second factor affecting the diversity of the local climate characteristics of Łódź in relation to less urbanized areas is anthropogenic pressure, the effects of which on climate change are concentrated in the central zone of the city. A significant amount of air pollutants accumulated there, which are a source of condensation of water vapor, combined with stronger convection over the city, caused by a higher temperature in its centre than on the outskirts, it affects the increase in the number of cloudy days in relation to neighbouring areas. The amount of solar energy reaching the city surface is therefore less than in the surrounding areas. Despite the smaller amount of solar energy, the area of Łódź is not cooler than the surrounding areas. The city produces a large amount of thermal energy, increasing the temperature of the air in the ground layers (its sources include, among others: heating, transport, electricity, technological processes in industry). As a result, in built-up areas, mainly in winter, the air temperature is higher than in non-built-up areas, creating an UHI. Due to the higher temperature and faster evaporation from hardened surfaces, the humidity in the city is about 10% lower than in undeveloped areas (although precipitation in Łódź is higher than in neighbouring areas).

More than half of the city is built-up and urbanized areas, with the largest part being residential areas [11]. Among the undeveloped areas, the largest percentage is still arable land, orchards, meadows, pastures (57%) − these areas are located mainly on the outskirts of the city. The areas of forest land and woodlands constitute a total of 28% of the area of undeveloped land.

The city is characterized by a regular architectural layout with a concentric-radiant spatial structure shaped as a result of natural conditions and historical and contemporary development processes. In its central parts, the streets are densely built with tenement houses creating typical street canyons. The increase in the building index significantly affects the thermal relations in the city. The most densely built-up area is the area of the city center of Łódź, where the development index reaches over 50%. The edge of the city is characterized by a building index of <10%.

Conditions conducive to the creation of an UHI may also be a low ventilation rate in the city. It is related, among others, to the height of the buildings shaping, among others, street canyons. Assessment of UHI in Łódź in 2022 revealed that intensity of the phenomenon for the downtown district is almost 7 times higher than the value obtained for the other areas [26]. An analysis of the UHI in Łódź in the years 2015–2018 carried out by Krawczyk et al. [27] showed, compared to the years 1997–1999, a more than 2-fold increase in the incidence of UHI ΔT >3°C at night in all seasons, although measurements were conducted only in 2 positions: city centre and Łódź-Lublinek (suburban area). The authors data show that a large degree of urbanization of the Łódź agglomeration along with the growth caused the extension of the phenomenon of the UHI to a larger area, and the positions that previously had high temperature contrasts (e.g., in Łódź-Lublinek) has now been absorbed into the range of UHI. Similar effect has been observed in other agglomerations due to urban sprawl [28]. The analysis carried out in this study covers the last 6 years (2014–2019), which were considered extremely warm. Although the model-generated temperature distribution shows less spatial differentiation than the literature research shows, it was found that the UHI phenomenon in Łódź is of a lasting nature.

Although the results of modeling are much less sensitive than the results of measurements, the days with the occurrence of the UHI phenomenon could be clearly determined. The most common temperature contrasts occurred in the summer months and in the early morning, evening and night hours. Generally, the marked contrasts were smaller than the measurement would indicate, but definitely the area of UHI was associated with dense development, which is consistent with the observations of other researchers [27,29,30].

These findings suggest a need for targeted interventions to mitigate UHI effects, particularly in the context of increasing climate warming and its potential to exacerbate heat stress in urban areas. Implementing strategies such as increasing green spaces, using reflective materials, and improving urban design could help reduce the impact of UHIs [31,32].

## CONCLUSIONS

The consistent presence and variability of the UHI phenomenon in Łódź over the 6-year period highlight significant challenges and opportunities for urban planning and public health, since it is primarily unfavourable for city dwellers causing heat stress, which adversely affects the physiological processes of the body and increases the morbidity, e.g., respiratory diseases [33,34].
